# HGT-ID: an efficient and sensitive workflow to detect human-viral insertion sites using next-generation sequencing data

**DOI:** 10.1186/s12859-018-2260-9

**Published:** 2018-07-17

**Authors:** Saurabh Baheti, Xiaojia Tang, Daniel R. O’Brien, Nicholas Chia, Lewis R. Roberts, Heidi Nelson, Judy C. Boughey, Liewei Wang, Matthew P. Goetz, Jean-Pierre A. Kocher, Krishna R. Kalari

**Affiliations:** 1 Division of Biomedical Statistics and Informatics, Department of Health Sciences Research, Mayo Clinic, Rochester, MN USA; 2 Department of Surgery, Mayo Clinic, Rochester, MN USA; 3 Division of Gastroenterology and Hepatology, Department of Internal Medicine, Mayo Clinic, Rochester, MN USA; 4 Department of Molecular Pharmacology and Experimental Therapeutics, Mayo Clinic, Rochester, MN USA; 5 Department of Medical Oncology, Mayo Clinic, Rochester, MN USA

**Keywords:** Horizontal gene transfer, Viral integration, Next-generation sequencing, Whole-genome sequencing, RNA-Seq – Cancer

## Abstract

**Background:**

Transfer of genetic material from microbes or viruses into the host genome is known as horizontal gene transfer (HGT). The integration of viruses into the human genome is associated with multiple cancers, and these can now be detected using next-generation sequencing methods such as whole genome sequencing and RNA-sequencing.

**Results:**

We designed a novel computational workflow, HGT-ID, to identify the integration of viruses into the human genome using the sequencing data. The HGT-ID workflow primarily follows a four-step procedure: i) pre-processing of unaligned reads, ii) virus detection using subtraction approach, iii) identification of virus integration site using discordant and soft-clipped reads and iv) HGT candidates prioritization through a scoring function. Annotation and visualization of the events, as well as primer design for experimental validation, are also provided in the final report. We evaluated the tool performance with the well-understood cervical cancer samples. The HGT-ID workflow accurately detected known human papillomavirus (HPV) integration sites with high sensitivity and specificity compared to previous HGT methods. We applied HGT-ID to The Cancer Genome Atlas (TCGA) whole-genome sequencing data (WGS) from liver tumor-normal pairs. Multiple hepatitis B virus (HBV) integration sites were identified in TCGA liver samples and confirmed by HGT-ID using the RNA-Seq data from the matched liver pairs. This shows the applicability of the method in both the data types and cross-validation of the HGT events in liver samples. We also processed 220 breast tumor WGS data through the workflow; however, there were no HGT events detected in those samples.

**Conclusions:**

HGT-ID is a novel computational workflow to detect the integration of viruses in the human genome using the sequencing data. It is fast and accurate with functions such as prioritization, annotation, visualization and primer design for future validation of HGTs. The HGT-ID workflow is released under the MIT License and available at http://kalarikrlab.org/Software/HGT-ID.html.

## Background

Horizontal gene transfer (HGT), or the transfer of genes between organisms in a manner other than traditional reproduction, was first described in 1928 when Frederick Griffith converted nonvirulent *Streptococcus pneumoniae* cells into infectious cells by exposing them to an extract made from virulent but dead *S. pneumoniae* cells [1]. Recently, scientists have begun to question whether HGT from microbes and viruses could play a role in the development of cancer [2, 3]. With the most recent estimate, nearly two million cases of cancer—roughly 18% of the global cancer burden—were thought to be attributable to infectious origins [4, 5]. Although most known carcinogenic pathogens in humans are believed to work by establishing persistent inflammation [6], some cancer-associated viruses integrate into the genome [7–9]. These integrations could potentially disrupt the genome like that of transposable elements [3]. For example, hepatitis B virus (HBV) integration is observed in more than 85% of hepatocellular carcinomas (HCCs), and copy-number variation significantly increases at HBV breakpoint locations, suggesting that integration of the virus induces chromosomal instability [10]. Also, recurrent integration events are associated with up-regulation of cancer-related genes, and having three or more HBV integrations is associated with reduced patient survival [10]. Similarly, various studies have reported integration of the human papillomavirus (HPV) in 80 to 100% of cervical cancers [11–13]; here, too, integration is associated with reduced survival [11], presumably because it disrupts coding regions important in the regulation of viral genes [14]. Merkel cell polyomavirus integration is found in 80 to 100% of Merkel cell carcinomas, a rare and aggressive form of skin cancer [15, 16]. Here, it is thought that truncation of the viral T-antigen protein complex, caused by integration, results in increased cell proliferation, leading to cancer [17]. Finally, in areas of Africa in which Burkitt’s lymphoma is endemic, Epstein-Barr virus (EBV) infection is found in nearly 100% of cases, and one hypothesis is that viral integration into the host genome contributes to the translocation involving the *MYC* oncogene that is responsible for this disease [18, 19].

Increasingly, researchers have been interrogating RNA-Seq data to determine whether the expression of viral sequences is associated with other types of cancer as well. Two recent studies have attempted to identify viral signatures in RNA sequencing data from many different types of cancers [20, 21]. These studies found that although HPV, HBV, and EBV signatures were associated with various types of cancer, including those mentioned above, no viral signatures were identified for common cancers such as breast, ovarian, and prostate cancer. Also, another study of 58 breast cancer transcriptomes found no significant viral transcription [22]. Notably, however, none of these findings exclude the presence of non-transcribed viral DNA in other common types of cancers. Thus, it is important to develop methods of interrogating both RNA-Seq and whole genome sequencing (WGS) data for potential viral insertion sites.

Existing methods for identifying viral integration sites are based on the subtraction approach, which removes mapped human reads and focuses on unmapped reads in the aligned bam files. For example, the VirusSeq software [23] was one of the first methods to identify potential viral integration events in RNA-Seq data based on subtraction analysis. VirusSeq was later outperformed by ViralFusionSeq [24], VirusFinder [25], and VirusFinder2 [26]. Among the above methods, VirusFinder2 is considered to have the best performance, achieved by applying the VERSE algorithm to customize the viral and host genomes in order to improve mapping rates [26]. Despite the resource-intensive reassembly and remapping of the reads, the sensitivity of VirusFinder2 is less than ideal, possibly due to the stringent hard thresholds chosen in the VERSE algorithm. Recently, the BATVI software [27] applied a k-mer aligner to achieve fast and accurate detection of viral integrations. However, we observed the drawback that most of the above algorithms use ad hoc read depths as cutoffs to select the candidate events. Hence, we designed a novel computational workflow, HGT-ID, to identify the integration of viruses into the human genome using sequencing data; the HGT-ID workflow utilizes a scoring function to select and prioritize the HGT candidates to achieve high sensitivity and specificity together with high efficiency. We compared our algorithm with VirusFinder2 and BATVI with a simulation dataset. The algorithm was also applied to multiple cancer datasets [10, 28–30] and was proved to have high sensitivity and specificity in detecting the HGT candidates compared to the existing software. For the convenience of downstream analysis, our HGT-ID software provides an integrated HTML report that includes prioritization of the candidate HGT events, visualization of the events and primers designed for future experimental validation.

## Implementation

**Fig. 1:**
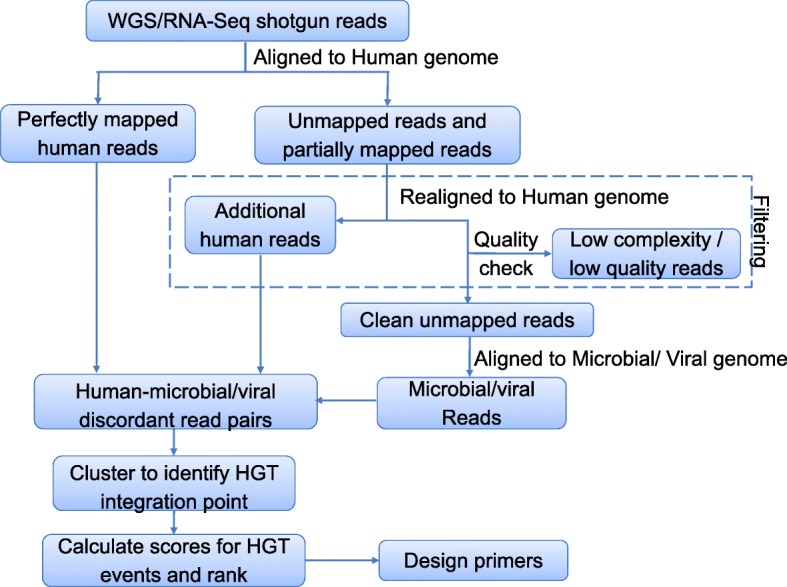
Overview of the HGT-ID workflow

HGT-ID follows a four-step procedure that includes the preprocessing of a previously aligned BAM file to the human genome, the detection of viral species with unmapped reads, identification of the viral integration sites as HGT candidates, and finally the priority score assignment by a scoring function (Fig. 1).

### Preprocessing

As input, HGT-ID requires paired-end next-generation sequencing (NGS) data in the standard BAM file format generated by any aligner using the human genome reference. Unmapped reads from the BAM file are extracted and then remapped to the human reference genome (hg19) using BWA-mem [31] to remove any additional human reads. Both mapped human and unmapped paired-end reads are filtered from further analysis. Only partially mapped read pairs, with one of the reads mapped to the human genome are collected as potential integrated viral reads for future HGT detection.

### Viral reads alignment

For the viral detection, we use the RefSeq Viral genome database [32] as the reference, which covers 6009 known species (ftp://ftp.ncbi.nih.gov/refseq/release/viral, as of March 2015) and is a reasonable collection of representative consensus sequences for different strains. Potential viral reads from the preprocessing step above are then aligned to the RefSeq viral reference genome using the BWA-mem software. After the viral alignment, read pairs with both ends mapped to viral species only are filtered. As direct evidence of viral integration, reads with one end mapped to the viral genome and other end assigned to the human genome are retained for further analysis. In order to remove low complexity sequence that is common in viral sequences and might affect the alignment, we calculate the sequence linguistic complexity (LC) score [33] of each read mapped to the viral genome. The recommended default threshold is 0.8, which is the upper range of LC scores of the low complexity and simple sequence of length 50-150 bp in the RepeatMasker [34]. Reads with LC scores < 0.8 are removed to improve both accuracy and efficiency. Low quality reads with mapping quality scores (MAPQ) below 20 are also removed, which ensures the mapping correctness with a *p*-value less than 0.01 for each kept read. The remaining discordant read pairs are considered as confident supporting reads for the viral integration step. Although we have set the default to recommended values, all the parameters listed in this section are customizable through the configuration files by the user.

### Viral integration site detection

**Fig. 2:**
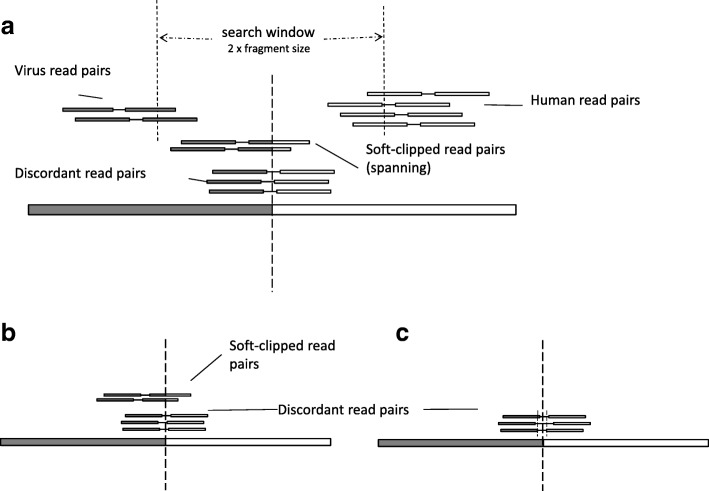
Diagram of HGT event and break point identification. **a** The searching starts with clustered discordant read pairs. Reads that fall within a search window of twice of the library size around the cluster are extracted. **b** If soft-clipped reads are available, an exact integration site can be inferred. **c** If only discordant read pairs are available, only an approximate integration site can be inferred

The viral integration sites are identified in a two-step process. First, for the discordant read pairs, HGT-ID clusters the human reads by their genomic location. The clusters then expand to both upstream and downstream directions recursively (default 500 bp, which is slightly larger than the size of the library fragments) until no more human reads from discordant read pairs can be recruited. For each cluster, a putative breakpoint is then estimated by taking the average of the start points of all reads in the cluster. The same procedure is also applied to the virus side to obtain a putative viral genomic breakpoint (Fig. 2a).

In the second step, HGT-ID scans for soft-clipped human reads around the putative breakpoint. The search window is centered at the breakpoint, spanning both upstream and downstream regions to match the size of the library fragments. Before each soft-clip read can be recruited into the read cluster, the soft clipped section is compared with the viral genome to remove spurious soft-clipped reads that do not belong to the virus. Among the cleaned reads, if there are soft-clipped reads that span through the breakpoint, a precise integration site can be inferred for the human side (Fig. 2b). Otherwise, the middle point of the clustered discordant read pairs is obtained as the approximate integration site (Fig. 2c). Similarly, on the viral side, the integration sites can be obtained by the same procedure described above.

### HGT candidate score function

The goal of HGT-ID is to identify high confident HGT events that are associated with high genomic instability. High confident HGT events tend to have high read coverage that supports the event against the background. On the other hand, false positive HGT events are indicative of a relatively low number of supporting reads that might occur due to random chimeric integration of fragments during sequencing [35]. Thus, the HGT-ID algorithm ranks the candidate events by applying a scoring function that compares the HGT supporting reads to the local background.

To estimate the local expected background for a given candidate event, first, the local coverage *N*_*local*_ is counted by including all the reads falling in a window that is centered at the breakpoint and spanning both upstream and downstream for the library fragment length. The local probability of a human read to randomly integrate with viral reads can be roughly estimated as *P*_*H*_ = *m*_*H*_/*N*_*local*_, where *m*_*H*_ is the number of human reads that are either split or spanning through the breakpoints. Similarly, for the integrated viral reads, we can calculate *P*_*V*_ = *m*_*V*_/*N*_*local*_, where *m*_*V*_ is the number of viral reads that are either split or spanning through the breakpoints. Then, the probability of supporting coverage generated by a random integration of human and viral reads should be proportional to the product of *P*_*H*_ and *P*_*V*_. The expected number of random discordant reads *count*_*bg*_ can then be estimated as:

$$ {count}_{bg}={P}_H\ast {P}_V\ast {N}_{local}={m}_H\ast {m}_V/{N}_{local} $$

The supporting coverage of the given candidate event (*count*_*sp*_) is calculated as the sum of discordant read pairs (*count*_*D*_), soft-clipped reads identified in human (*count*_*sch*_) and viral (*count*_*scv*_) bam files respectively, i.e.,

$$ {count}_{sp}={count}_D+{count}_{sch}+{count}_{scv} $$

And the prioritizing score of the given candidate events can be calculated as

$$ score={count}_{sp}-{count}_{bg} $$

If the score is negative for a given candidate event, HGT-ID will still report it, but the event should be taken as false positive.

### Primers design for experimental validation

The HGT candidates can be typically validated by polymerase chain reaction (PCR) experiments. The HGT-ID workflow thus provides a primers design function, which designs oligonucleotide primers that flank the detected viral integration sites (a sample report together with sample results are provided in the website http://kalarikrlab.org/Software/HGT-ID.html) using Primer3 [36]. The best primer candidates are chosen by optimizing primer length, melting temperature, and binding tendencies in addition to product length. Only the top-scoring primer pair from each side of the viral integration site is returned to the user. These four primers make two PCR products, which can be used to validate the human boundaries of the viral integration site; they are intended to be utilized in a standard PCR experiment to confirm findings from the HGT-ID workflow. If the viral sequence integrated into the human genome is short enough (< 5 kb), the user can use the forward primer for the first product and the reverse primer for the second product to amplify the entire integration event.

### Visualization and report

For each sample processed through the workflow, the method provides a comprehensive report in HTML with annotation, visualization and customer primer design for experimental validation (a sample report is provided in the website http://kalarikrlab.org/Software/HGT-ID.html). Beyond the details of each candidate event and the designed primers, the report also gives circos plots to visualize the location and coverage of each event in both human genome and viral genome.

### Generation of simulated data

We used a simulator program provided by the ViralFusionSeq [24] package (simulate-viralfusion.pl) to generate a simulated FASTA file. In the simulated genome, the human chromosomes 1–4 (hg19) were randomly infected by HPV strain (HPV18 9,626,069). We used the option as “--virus-block-len 400 –lowvirus 75 --high-virus 100”. The resulting simulated genome contained 249 HGT integration sites, based on the simulation report. Next, we generated 40× coverage whole genome sequencing simulated data with a 300 bp library fragments size and 101 bp read length using the Wgsim simulator [37] with default parameters. Specifically, we generated 20 million paired-end reads from the simulated genome with the options “-N 10000000 -1 101 -2 101”. It should be noted that Wgsim is able to simulate genomes with SNPs and insertion/deletion (INDEL) polymorphisms, and simulate reads with uniform substitution sequencing errors [37]. From these simulated WGS data, we generated additional sequencing datasets by downsampling to 75% (30X), 50% (20X), 25% (10X), 10% (4X) and 5% (2X) of the original data, respectively.

### Sequencing datasets used to validate HGT-ID

To test and validate the performance of HGT-ID workflow, we have applied the HGT-ID algorithm to several publicly available NGS datasets, including both WGS data and RNA-Seq (Table 1).

**Table 1:** Sample sets that were used to validate the performance of HGT-ID

Sample Set	Possible Virus	Data type	No. of Samples	Ref
1. Cervical cell lines and cervical carcinoma	Human papillomavirus	WGS	4 WGS	[28]
2. Hepatocellular carcinoma	Hepatitis B virus	WGS	13 WGS	[10]
3. TCGA Breast invasive carcinoma	NA	WGS	220 WGS	[29]
4. Hepatocellular carcinoma	Hepatitis B virus	WGS + RNA-Seq	7 WGS + 7 RNA-Seq	https://cancergenome.nih.gov/

## Results

### HGT event detection in simulated data

We compared the performance of HGT-ID, BATVI, and VirusFinder2 with the simulated data. In this comparison, if an integration site falls within the distance of the library fragment size (which was 300 bp in this simulation data) from the actual inserted site, it was counted as true positive.

Table 2 provides the performance comparison of HGT-ID, BATVI, and VirusFinder2 with the simulated data at different sequence depth coverage. HGT-ID demonstrated the highest sensitivity among all three algorithms. HGT-ID detected all of the true positives (TP) in the datasets with coverage of 4X or more, and it was still highly sensitive at the very low coverage of 2X. BATVI demonstrated both lower sensitivity and lower specificity than did HGT-ID in the datasets with coverage of more than 4X. VirusFinder2 demonstrated the lowest false positive (FP) rate in the simulation data; however, it had the lowest sensitivity, which also dropped substantially with coverage of 4X or less.

**Table 2:** Performance comparison of HGT-ID, BATVI and VirusFinder2

Coverage	Simulated data *N* = 249					
	HGT-ID		BATVI		VirusFinder2	
	TP	FP	TP	FP	TP	FP
40	249	16	244	52	234	3
30	249	16	244	40	234	1
20	249	14	246	24	220	4
10	249	8	246	11	206	2
4	249	6	230	6	121	1
2	237	20	190	2	40	2

**Fig. 3:**
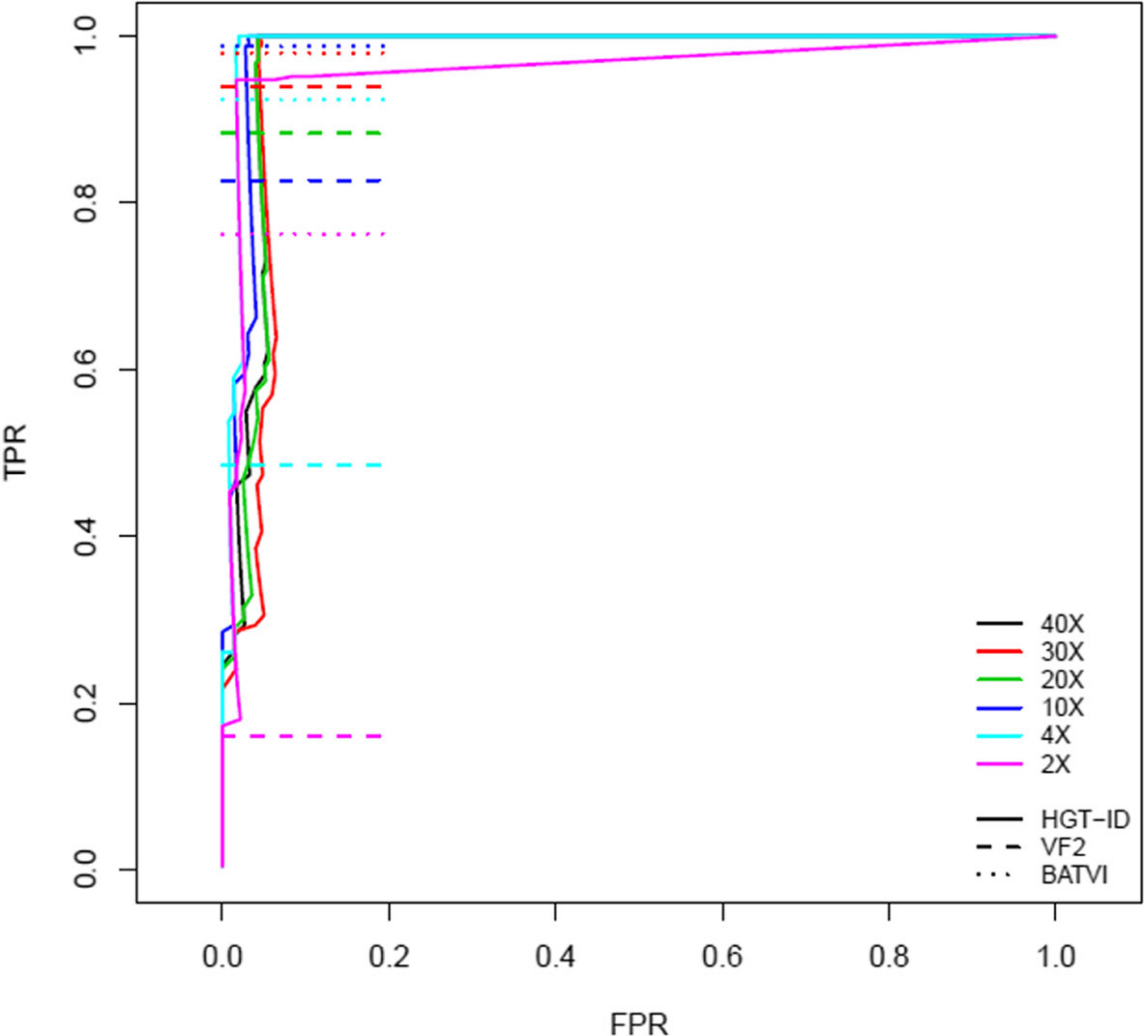
ROC curve of the simulation data with different coverages of HGT-ID. Different color lines showed different coverages. The false positive ratio (FPR) was calculated as the ratio of the number of false positives and the number of total identified HGT events. The true positive rate (TPR) was calculated as the ratio of the number of true positives and the number of total positives. The coverages were down-sampled from 40X to 30X, 20X, 10X, 4X and 2X, respectively

From the performance evaluation in Table 2, we recommend using at least 4X coverage to ensure optimal performance of HGT-ID. Figure 3 illustrates the ROC of HGT-ID across different coverages, which also confirmed the optimal usage of 4X and above. ROC curves (Fig. 3) as well as the distribution of scores (a sample report together with sample results are provided in the website http://kalarikrlab.org/Software/HGT-ID.html) of HGT events indicated that the optimal cutoff scores across different coverages was 0. It is noted that the performance evaluations of HGT-ID were based on this cutoff if not otherwise stated.

Different color lines illustrate different coverages. The false positive ratio (FPR) was calculated as the ratio of the number of false positives and the number of total identified HGT events. The true positive rate (TPR) was calculated as the ratio of the number of true positives and the number of total positives. The coverages were down-sampled from 40× to 30X, 20X, 10X, 4X and 2X, respectively.

### HPV detection in WGS data from cervical carcinoma samples and cell lines

We applied the HGT-ID workflow to a publicly available WGS dataset (SRA180295) with at least 30× coverage containing four HPV-positive samples: two HPV-positive cell lines (SiHa and HeLa) and two cervical carcinomas (T4931 and T6050) [28] (Table 3). Hu and co-authors generated WGS data for the four HPV samples and identified integration sites with experimental validation. They subsequently validated the integration sites with Sanger sequencing. Using the default parameters, HGT-ID detected the same 11 integration sites identified in the original publication (Table 3) with 1~ 3 bp difference because of the approximation of the algorithm. All 11 identified integration sites were either in the intron or the intergenic region. Some integration breakpoints that we detected in the human genome would be approximated close but not identical to the experimentally validated breakpoints due to the lack of soft-clip reads to refine the precise location in the two-step procedure we used to identify integration sites (see Methods for details). To compare HGT-ID’s performance with a similar viral integration site detection program, we also processed the same data with VirusFinder 2.0, using the default parameters. VirusFinder 2.0 was able to only detect 6 of the 11 integration sites identified in the original article. All detected integration events were scored high by HGT-ID except one in the T4931 cell line, due to less discordant supporting reads. As an example, the final HTML report generated by HGT-ID with details for the HeLa cell lines can be found in the website (http://kalarikrlab.org/Software/HGT-ID.html).

**Table 3:** All 11 viral integration sites identified in whole genome sequencing data from two HPV-positive cell lines (SiHa and HeLa) and two cervical carcinomas (T4931 and T6050) using HGT-ID

Sample ID (coverage)	Affected Gene	Function of integration site	Integrated Position	Score	Reported and validated^a^	Identified by VirusFinder 2.0
HELA (40x)	CCAT1	intron	chr8 128,230,630	1273.7	yes	yes
	CCAT1	upstream	chr8 128,233,368	121.2	yes	no
	CCAT1	upstream	chr8 128,234,256	180.3	yes	no
	CCAT1	upstream	chr8 128,241,549	235.7	yes	yes
SIHA (37×)	KLF12	downstream	chr13 74,087,563	158.0	yes	yes
	KLF12	downstream	chr13 73,788,864	136.4	yes	yes
T4931 (41×)	GLI2	intron	chr2 121,670,164	2.4	yes	yes
	GLI2	intron	chr2 121,687,141	213.4	yes	no
	GLI2	intron	chr2 121,688,179	48.9	yes	no
T6050 (42×)	KLF12	downstream	chr13 74,230,820	305.1	yes	no
	KLF12	downstream	chr13 74,231,436	342.2	yes	yes

^a^Reported and validated in the original paper [28]

As shown in Table 3, the HGT events in HELA cervical cancer cell lines were observed in the upstream region of the long non-coding RNA CCAT1. A recent study indicated that CCAT1 might promote proliferation and inhibit apoptosis of cervical cancer cells by activating the Wnt/β-catenin pathway [38]. The HGT-ID workflow also identified an HGT candidate downstream of KLF12, a tumor suppressor gene [39, 40], in both the SIHA cervical cancer cell line and a tumor sample. HGT-ID also identified another target gene GLI2 that is important in the Hedgehog pathway and is known to be critical in tumorigenesis [41].

### HBV detection in liver cancer samples

#### Dataset I

We tested the performance of HGT-ID by applying the algorithm to 13 HBV-positive HCC samples [10] with default settings and requiring at least two discordant read pairs as direct evidence. In total, we detected 83 viral integration sites, of which 67 events had a prioritization score larger than or equal to 10.

We compared our results with the original paper, which provided experimental validation for 22 randomly selected viral integration sites from 13 tumor samples. HGT successfully identified 18 of these 22 experimentally identified viral integration sites, with all 18 scoring 10 or higher (Table 4, http://kalarikrlab.org/Software/HGT-ID.html). The four missing events have no discordant human-viral read pairs, resulting in their being filtered out from our candidate events. Further investigation of the missing events revealed that these four events consisted of very short viral insertions (~ 60 bp) that were smaller than the read length (90 bp). Thus, there were no complete viral reads to form a discordant pair to pass the minimal evidence required for an HGT candidate event in HGT-ID.

**Table 4:** Validation of the integration sites in HPV data

Sample ID and coverage	Affected genes	Function of integration site	Integration breakpoints in the human genome	Integration breakpoints in HBV virus	Score	Identified by HGT-ID?
145 T (37×)	CCNE1	intron	chr19: 30303492	1053	87.2	yes
	CCNE1	intron	chr19: 30303498	1819	87.2	yes
177 T (43×)	SENP5	intron	chr3: 196625752*	1827*	–	no
180 N (121×)	FN1	intron	chr2: 216280279	1822	11.9	yes
186 T (36×)	KMT2B	exon	chr19: 36214005	2448	206.2	yes
	KMT2B	exon	chr19: 36214017	1605	206.2	yes
198 T (34×)	TERT	intron	chr5: 1269387	821	137.5	yes
	TERT	intron	chr5: 1269405	1950	137.5	yes
26 T (66×)	DUX4	intron	chr18: 107920*	670*	–	no
200 T (32×)	CCNE1	exon	chr19: 30315003	1798	51.4	yes
	CCNE1	downstream	chr19: 30315365	316	222.751	yes
268 T (34×)	CCNE1	upstream	chr19: 30298787	1931	155.2	yes
	TERT	intron	chr5: 1291758	3175	134.3	yes
	TERT	intron	chr5: 1292403	354	134.3	yes
43 T (33×)	SENP5	intron	chr3: 196625710*	1910*	–	no
46 T (32×)	TERT	upstream	chr5: 1295367	751	34.4	yes
70 T (114×)	KMT2B	exon	chr19: 36212331	1931	1015.6	yes
	KMT2B	exon	chr19: 36212311	227	1015.6	yes
71 T (32×)	SENP5	intron	chr3: 196625776*	417*	–	no
	KMT2B	intron	chr19: 36213141	1884	10	yes
	KMT2B	intron	chr19: 36213136	619	10	yes
95 T (35×)	KMT2B	exon	chr19: 36212564	2240	27.3	yes

Eighteen of 22 previously experimentally validated viral integration sites identified in sequencing data from 13 HBV-positive hepatocellular carcinoma samples using the HGT-ID algorithm. Integration breakpoints of the four missing events (noted with *) were obtained from the original publication [10]

To further validate the specificity of HGT-ID, we downloaded five samples (106 T, 117 N, 126 N, 203 T, and 73 T) from the same data set, which contained false positive HGT events that the original publication identified as candidates but failed to validate. HGT-ID did not pick up any negative events reported in these five samples. While this did not indicate that all other candidate events identified by HGT-ID were true positives due to the limited validation available, HGT-ID had exhibited great performance in accuracy. Overall HGT-ID accurately identified and confirmed 23/27 events (85.2%). On the contrary, VirusFinder 2.0 identified only 16 of 22 (72.7%) [26]. Once again, HGT-ID showed a higher sensitivity, though specificity could not be calculated because of the lack of validation data. In-depth investigation of the four events missed by the HGT-ID workflow determined that the candidates did not meet the minimum requirement of 2 read pairs; hence they likely did not meet the detection criteria.

#### Data set II

To check the performance of HGT-ID in both DNA and RNA sequencing data, we processed paired WGS (100 bp PE) and RNA-Seq samples (50 bp PE) from seven TCGA hepatocellular carcinoma (HCC) samples that were originally contributed by the Mayo Clinic. The summary of NGS reads for WGS and RNA-Seq platforms for these seven tumor-normal pairs are described in the website (http://kalarikrlab.org/Software/HGT-ID.html). The HGT algorithm was applied to all of the samples with the default settings, and integration events with a score > 10 were reported for both DNA and RNA samples.

Using WGS tumor data, we identified Hepatitis B virus (HBV) integration events in six out of seven TCGA HCC tumors (a sample report together with sample results are provided in the website http://kalarikrlab.org/Software/HGT-ID.html). In addition, HGT-ID workflow identified zero HGT events and a total of 42 HGT candidates in liver normal and tumor samples, respectively. Investigating RNA-Seq data from the same seven TCGA liver samples, the HGT-ID workflow, identified eight HGT candidates in tumors and six HGT events in normal adjacent samples. Comparison of the HGT sites from WGS and RNA-Seq data has identified an overlap of six events in TCGA liver tumors (Table 5). Details of the 62 HGT events detected in the seven samples are listed the website (http://kalarikrlab.org/Software/HGT-ID.html).

**Table 5:** Viral HGT events detected by HGT-ID algorithm between paired TCGA HCC tumor and normal samples via WGS and RNA-Seq datasets

Sample ID	WGS-T	WGS-N	RNA-T	RNA-N	Common HGT
TCGA-BW-A5NP	11	0	2	NA	0
TCGA-CC-5262	3	0	2	NA	2
TCGA-CC-A1HT	5	0	2	NA	2
TCGA-DD-A1EH	0	0	0	2	NA
TCGA-DD-A1EI	2	0	1	2	1
TCGA-DD-A1EL	17	0	1	2	1
TCGA-G3-A3CK	4	0	0	NA	0

T stands for primary solid tumor and N for matched solid normal tissue. Only 3 of the 7patients had RNA-Seq data for matched normal tissue. The “Common HGT” column contains the number of events that were identified in both WGS and RNA-Seq for the primary tumor (T)

Application of the HGT-ID workflow to the two HCC data sets has identified several HGT integration sites of HBV in liver cancer samples [10]. The affected genes included TERT, which plays a significant role in cancer cell immortality, and the mutation in its promoter region which is one of the most frequent alterations in HCC [42, 43]. Other genes like CCNE1, SENP5, FN1, KMT2B, and DUX4 were alsoidentified by HGT-ID; these genes were previously reported to be associated with tumorigenesis or cancer invasion [44–49].

### Viral integration detection in WGS data from breast cancer samples

The HGT-ID algorithm was applied to WGS data from 220 breast cancer samples collected by The Cancer Genome Atlas (TCGA) (a sample report together with sample results are provided in the website http://kalarikrlab.org/Software/HGT-ID.html). No exogenous viral integration events were detected in these samples. Our results are consistent with the results reported in previous studies [20, 21] and consistent with our findings using RNA-Seq data.

### Software performance evaluation

We compared the computational performance of our workflow with VirusFinder2 (VERSE algorithm). Using the HPV dataset as an example, HGT-ID used on average 14% of the time required by VirusFinder2 with VERSE when running on the same machine with default settings (a sample report together with sample results are provided in the website http://kalarikrlab.org/Software/HGT-ID.html). As an example, for the HELA cell line sample, HGT-ID used only 4.3 h while VirusFinder2 with VERSE used 23.4 h. BATVI was not able to finish processing any of the four cervical cell line dataset in our system. Further, we compared the running time on the smaller simulation datasets for all three algorithms (a sample report together with sample results are provided in the website http://kalarikrlab.org/Software/HGT-ID.html). HGT-ID demonstrated the fastest processing on the simulation datasets with highest coverage. The fast and accurate identification of HGT events by the HGT-ID workflow is primarily helpful in elucidating the effect of viral gene horizontal transfer on tumorigenesis and other diseases.

## Discussion

In this study, we present the HGT-ID workflow, which detects the viral integration sites in the human genome. The HGT-ID workflow is comprehensive and fully automated from the initial pre-processing step to the viral integration site detection, prioritization, and downstream visualization as well as primer design for validation. This workflow enables unbiased detection of viral integration events against the RefSeq viral database [32] without knowing the species in advance. Unlike VirusFinder2 and BAVTI [26, 27], HGT-ID reports both the viral names and the integration sites from multiple viral species/strains simultaneously, which will be convenient for co-infection analysis.

We have shown both higher sensitivity and specificity than the recent BATVI software. We also demonstrated better sensitivity than VirusFinder2 with comparable specificity across different coverage depths in both the simulation data set and the cancer data sets. Unlike other algorithms that directly use read counts as the cut-off threshold, HGT-ID calculates a score for each candidate HGT event making use of both supporting reads and background reads. The scores are used to rank the candidate HGT events. The higher the score, the more confident the HGT event tends to be. We suggest an empirical cutoff score of 10 for use with cancer data sets. By default, HGT-ID will output all candidate HGT events, ranked in order of decreasing score.

We applied the HGT-ID workflow to publicly available large cancer cohorts, such as TCGA, to study HCC and breast cancer. We have shown the applicability of the tool in HCC samples where we have both WGS and RNA-Seq data sets available. We have surveyed the breast cancer data set using our workflow and did not find any evidence of HGTs. Among all of the events detected by HGT-ID in this report, we found about ~ 50% of events occured in highly repetitive regions masked by RepeatMasker [34], like microsatellite, long terminal repeat (LTR), short interspersed elements (SINE) and Alu elements. In general, these regions are known to be related to genome instability and cancer development. It should be noted that in the simulation study, most of our small number of false positives (~ 5% of total reported events) were from such regions. As a precaution to users, we currently annotate the results if the candidate event is located in a RepeatMasker region (please refer to the sample output at the software download page).

We compared the computational performance of our workflow with VirusFinder2 (VERSE algorithm). VERSE intends to capture the consensus sequence to cover possible mutation in the virus by performing de-novo assembly. However, executing the VirusFinder2 with the VERSE algorithm is very time-consuming. Using the HPV dataset as an example, HGT-ID used on average only 14% of the time required by VirusFinder2 with VERSE when running on the same machine with default settings (a sample report together with sample results are provided in the website http://kalarikrlab.org/Software/HGT-ID.html). In addition, for the HELA cell line sample, HGT-ID used only 4.3 h while VirusFinder2 with VERSE used 23.4 h. To study other cancers or diseases with WGS or RNA-Seq data, the researchers can easily download the workflow and process the data through the HGT-ID to detect additional HGT candidates. The user manual and workflow are available to download. The fast and accurate identification of HGT events by the HGT-ID workflow is primarily helpful in elucidating the effect of viral gene horizontal transfer on tumorigenesis and other diseases.

As limited by the design of the algorithm, which requires discordant read pairs to start clustering, HGT-ID can only be applied to paired-end sequencing reads. HGT-ID applies a subtraction strategy to focus on unmapped reads that don’t belong to the human genome. Viral species are identified by aligning against the RefSeq viral genome database; thus, novel viral species will not be detected. We recommend updating the viral reference genome database to the latest NCBI RefSeq version before running HGT-ID workflow. Viral genomes are known for high mutation rates, which might prevent some of the sequences from being mapped to the reference viral genome. This problem can be partially solvedd by adjusting the aligner parameter to tune it to a more sensitive mode.

HGT-ID workflow was implemented in Perl and Bash programming language and has been tested on various Linux platforms. It depends on several third-party tools, including SAMtools [50], BedTools [51], in addition to the BWA-mem as mentioned earlier [30]. HGT-ID provides visualization of the detected integration sites using the RCircos [52] method. All of these tools are publicly available and are also packaged as part of the HGT-ID package. The software package together with an example is available at http://kalarikrlab.org/Software/HGT-ID.html.

## Conclusion

HGT-ID is a novel computational workflow to detect the integration of viruses in the human genome using the sequencing data. It is fast and accurate with functions such as prioritization, annotation, visualization and primer design for future validation of HGTs. The pipeline is now applied in several research and clinical projects at the Mayo Clinic for cancers that are associated withviruses. In the future, we plan to extend the application to detect bacterial HGT as well.

## Availability and requirements

- **Project name**: HGT-ID
- **Project homepage**: http://kalarikrlab.org/Software/HGT-ID.html
- **Operating system(s):** Linux or VM
- **Programming language**: PERL, JAVA, R and BASH
- **Other requirements**: none
- **License:** Open Source (MIT license)
- **Any restrictions to use by non-academics**: none

## Abbreviations

EBV: Epstein-Barr virus
HBV: Hepatitis B virus
HCC: Hepatocellular carcinomas
HGT: Horizontal gene transfer
HPV: Human papillomavirus
NGS: Next-generation sequencing
PE: Paired-end
ROC: Receiver operating characteristic curve
TCGA: The Cancer Genome Atlas
WGS: Whole-genome sequencing
